# Foresight 2035: a perspective on the next decade of research on the management of *Legionella* spp. in engineered aquatic environments

**DOI:** 10.1093/femsre/fuaf022

**Published:** 2025-05-27

**Authors:** Frederik Hammes, Marco Gabrielli, Alessio Cavallaro, Antonia Eichelberg, Sofia Barigelli, Melina Bigler, Sebastien P Faucher, Hans P Füchslin, Valeria Gaia, Laura Gomez-Valero, Marianne Grimard-Conea, Charles N Haas, Kerry A Hamilton, Hannah G Healy, Yann Héchard, Tim Julian, Laurine Kieper, Ursula Lauper, Xavier Lefebvre, Daniel Mäusezahl, Catalina Ortiz, Ana Pereira, Michele Prevost, Hunter Quon, Siddhartha Roy, Ana R Silva, Émile Sylvestre, Lizhan Tang, Elliston Vallarino Reyes, Paul W J J van der Wielen, Michael Waak

**Affiliations:** Eawag, Swiss Federal Institute of Aquatic Science and Technology, 8600 Dübendorf, Switzerland; Eawag, Swiss Federal Institute of Aquatic Science and Technology, 8600 Dübendorf, Switzerland; Eawag, Swiss Federal Institute of Aquatic Science and Technology, 8600 Dübendorf, Switzerland; Eawag, Swiss Federal Institute of Aquatic Science and Technology, 8600 Dübendorf, Switzerland; Laboratory of Environmental and Applied Microbiology, Department of Chemistry, Biology and Biotechnology, University of Perugia, 06123 Perugia, Italy; Swiss Tropical and Public Health Institute, 4123 Allschwil, Switzerland; University of Basel, 4001 Basel, Switzerland; Department of Natural Resource Sciences, McGill University, Sainte-Anne-de-Bellevue, QC H9X 3V9, Canada; Zurich Cantonal Laboratory, 8032 Zürich, Switzerland; National Reference Center for Legionella, Institute of Laboratory Medicine , Ente Ospedaliero Cantonale, 6500 Bellinzona, Switzerland; Institut Pasteur, Université Paris Cité, Biologie des Bactéries Intracellulaires, CNRS UMR 6047, 75015 Paris, France; Department of Civil, Geological and Mining Engineering, Polytechnique Montréal, 83T1J4 Montreal, Canada; Department of Civil, Architectural and Environmental Engineering, Drexel University, PA 19104 Philadelphia, United States; School of Sustainable Engineering and the Built Environment, Arizona State University, AZ 85281 Tempe, United States; The Biodesign Center for Environmental Health Engineering, 1001 S McAllister Ave, Tempe, AZ 8528, United States; Department of Environmental Health, Harvard T.H. Chan School of Public Health, MA 02115 Boston, United States; Laboratoire Ecologie et Biologie des Interactions, Université de Poitiers, 86073 Poitiers, France; Eawag, Swiss Federal Institute of Aquatic Science and Technology, 8600 Dübendorf, Switzerland; Swiss Tropical and Public Health Institute, 4123 Allschwil, Switzerland; Institute for Molecular Microbiology and Virology, Medical Faculty and University Hospital Carl Gustav Carus, TUD Dresden University of Technology, 01307 Dresden, Germany; New York State Department of Health, NY 12223 Albany, United States; Department of Mechanical Engineering, Polytechnique Montréal, 83T1J4 Montreal, Canada; Swiss Tropical and Public Health Institute, 4123 Allschwil, Switzerland; University of Basel, 4001 Basel, Switzerland; Department of Civil, Geological and Mining Engineering, Polytechnique Montréal, 83T1J4 Montreal, Canada; LEPABE – Laboratory for Process Engineering, Environment, Biotechnology and Energy, Faculty of Engineering, University of Porto, 4200-465 Porto, Portugal; ALiCE – Associate Laboratory in Chemical Engineering, Faculty of Engineering, University of Porto, 4200-465 Porto, Portugal; Department of Civil, Geological and Mining Engineering, Polytechnique Montréal, 83T1J4 Montreal, Canada; School of Sustainable Engineering and the Built Environment, Arizona State University, AZ 85281 Tempe, United States; The Biodesign Center for Environmental Health Engineering, 1001 S McAllister Ave, Tempe, AZ 8528, United States; Department of Environmental Sciences, Rutgers University, NJ 08901 New Brunswick, United States; LEPABE – Laboratory for Process Engineering, Environment, Biotechnology and Energy, Faculty of Engineering, University of Porto, 4200-465 Porto, Portugal; ALiCE – Associate Laboratory in Chemical Engineering, Faculty of Engineering, University of Porto, 4200-465 Porto, Portugal; Sanitary Engineering, Delft University of Technology, 2628 CN Delft, the Netherlands; Eawag, Swiss Federal Institute of Aquatic Science and Technology, 8600 Dübendorf, Switzerland; Department of Natural Resource Sciences, McGill University, Sainte-Anne-de-Bellevue, QC H9X 3V9, Canada; KWR Water Research Institute, 3433 PE Nieuwegein, the Netherlands; Department of Microbiology, Radboud Institute of Biological and Environmental Science, Faculty of Science, Radboud University, 6525AJ Nijmegen, the Netherlands; Department of Civil and Environmental Engineering, Norwegian University of Science and Technology, Trondheim 7031, Norway; Department of Infrastructure, SINTEF Community, Trondheim 7031, Norway

**Keywords:** *Legionella*, Legionnaires’ disease, legionellosis, building plumbing, opportunistic pathogens, waterborne disease

## Abstract

The disease burden from *Legionella* spp. infections has been increasing in many industrialized countries and, despite decades of scientific advances, ranks amongst the highest for waterborne diseases. We review here several key research areas from a multidisciplinary perspective and list critical research needs to address some of the challenges of *Legionella* spp. management in engineered environments. These include: (i) a consideration of *Legionella* species diversity and cooccurrence, beyond *Legionella pneumophila* only; (ii) an assessment of their environmental prevalence and clinical relevance, and how that may affect legislation, management, and intervention prioritization; (iii) a consideration of *Legionella* spp. sources, their definition and prioritization; (iv) the factors affecting Legionnaires’ disease seasonality, how they link to sources, *Legionella* spp. proliferation and ecology, and how these may be affected by climate change; (v) the challenge of saving energy in buildings while controlling *Legionella* spp. with high water temperatures and chemical disinfection; and (vi) the ecological interactions of *Legionella* spp. with other microbes, and their potential as a biological control strategy. Ultimately, we call for increased interdisciplinary collaboration between multiple research domains, as well as transdisciplinary engagement and collaboration across government, industry, and science as the way toward controlling and reducing *Legionella-*derived infections.

## Teaser

A review of important research topics related to the control of *Legionella* spp. in engineered ecosystems, with a specific focus on critical knowledge gaps and future research opportunities.

## Introduction

**Figure 1.**
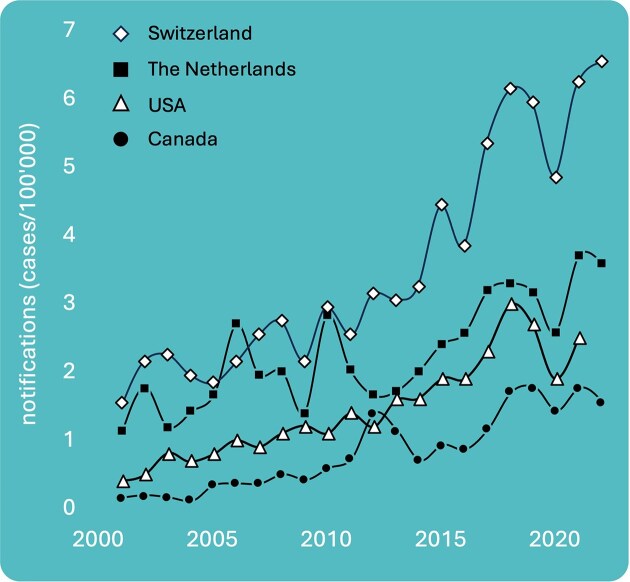
Reported Legionnaires’ disease cases during the last two decades in Switzerland, the Netherlands, the USA, and Canada. Data obtained from www.bag.admin.ch, www.ecdc.europa.eu/en, www.cdc.gov, www.diseases.canada.ca.

*Legionella* is a genus containing opportunistic pathogenic species that inhabit a range of natural and engineered environments, which are often closely entwined with everyday life, such as building plumbing systems, cooling towers, and thermal spas (NASEM 2020). There, they survive in polymicrobial biofilms and are believed to multiply predominantly inside diverse protists (Boamah et al. 2017, Barbosa et al. 2024). Following dispersal from biofilms into water, transmission to humans occurs mostly through inhalation of contaminated aerosols. Upon inhalation, pathogenic *Legionella* species cause infections in humans that manifest typically as severe pneumonia known as Legionnaires’ disease or milder flu-like Pontiac fever (Hamilton et al. 2018), while multiple other nontypical clinical pulmonary and extrapulmonary outcomes have been recorded (Muder and Yu 2002). In industrialized countries the disease burden from *Legionella*-derived infections ranks amongst the highest waterborne diseases (Fastl et al. 2020, McMullen et al. 2024). Despite decades of intense research that has been translated into legislation and management strategies, notification rates continue to increase worldwide (Fig. 1) (Moffa et al. 2023, Fischer et al. 2023a, Pareek et al. 2024).

Several studies argue that the actual incidence rates of Legionnaires’ disease are grossly underestimated, by factors as high as 2–10-fold (Cassell et al. 2019, McMullen et al. 2024). Increasing notification rates are partially ascribed to increased awareness and improved diagnostics (Graham et al. 2023), but also to aging populations, building practices, air quality (Yu et al. 2024), healthcare systems, legislation, demographics, water quality, aerosol sources, and climate change (Walker 2018). Moreover, different countries/regions evidently show different disease notification trends (e.g. Campese et al. 2011, Fischer et al. 2020), which presents an opportunity for researchers to try to untangle the underlying variables contributing to these trends.

A multidisciplinary group of international researchers representing different *Legionella*-related scientific fields gathered in Switzerland in June 2024 to map out the current state of the science with respect to *Legionella* spp. epidemiology, risk, management and ecology, and specifically explore the main research needs and opportunities for the coming years. Here, we report and expand on some of the key outcomes and perspectives from the meeting. This is not a consensus report, and it does not cover all aspects of *Legionella* spp. research; rather, we reflected the diverse opinions, uncertainties, and open questions on a subset of important topics on *Legionella* spp. management in the engineered aquatic environment.

## Underappreciated and unexplored *Legionella* spp. diversity

Scientific reporting frequently (and erroneously) generalizes *Legionella* spp. behavior (e.g. infectivity, growth, persistence, and stress response) as belonging to a singular, similar group (e.g. “*Legionella* are bacteria that*…*”). The genus *Legionella* is, in fact, highly diverse; current literature describes 77 species/subspecies, of which 74 are recognized with International Code of Nomenclature of Prokaryotes status (an updated species list with information regarding clinical association is available at: DOI: 10.5281/zenodo.11072744; LeCo Project 2024). This number most likely underestimates the actual number of species. In fact, a recent pangenomic analysis, including both genomes from isolated strains and metagenome-assembled genomes, advocates for moving *Legionella* taxonomy beyond the phenotypical criteria of isolated organisms and revealed the presence of at least 54 still uncultivated species belonging to the family *Legionellaceae*, of which 21 are highly related to *Legionella pneumophila* (Gabrielli et al. 2024). Moreover, amplicon sequencing surveys have highlighted that *Legionellales* bacteria are likely to include an even higher number of genera (Graells et al. 2018). In this section, we consider *Legionella* spp. diversity from an environmental and ecological perspective, and its implications for *Legionella* spp. management.

The historic clinical focus, as well as a recognized methodological bias toward *L. pneumophila* detection and isolation (e.g. Lee et al. 1993, Chambers et al. 2021), had as a result that the environmental diversity of *Legionella* spp. has largely been neglected and underappreciated. However, recent studies challenge this perspective, revealing not only heterogeneity across different environments, but also, critically, the cooccurrence of different *Legionella* species within the same environment. For example, Dilger et al. (2018) reported a large biogeographical investigation of cultivable *Legionella* spp. in Germany, with up to 42% of isolates in some regions as *Legionella* non-*pneumophila* species. Even more pronounced, a study in the Netherlands found that only 3.1% of the 206 buildings that were positive for *Legionella* spp. were also positive for *L. pneumophila* (van der Lugt et al. 2019). Moreover, genus-specific polymerase chain reaction (PCR) and subsequent sequencing revealed that drinking water sampled at treatment plants in the Netherlands consistently contained *Legionella* spp. and that 70% of the sequences belonging to not-yet-cultivated species were detected at water temperatures below 15°C (Wüllings and van der Kooij 2006), a drastically lower temperature than what is commonly used for *Legionella* spp. culturing. Changes in environmental conditions reflect, also, on which *Legionella* species are present and cooccurring in an environment. Girolamini et al. (2022) observed cooccurrence and community shifts of various *Legionella* species in hospital water treated with high temperature and disinfection. Lesnik et al. (2016) tracked *Legionella* spp. from source to tap in a drinking water system using genus-specific primers and demonstrated differences in *Legionella* taxa based on sampling location and water temperature, with coexistence of up to 14 unique phylotypes. Cavallaro et al. (2023) showed the presence of up to 30 unique *Legionella* spp. amplicon sequence variants based on 16S rRNA gene amplicon sequencing occurring in shower hose biofilms from the same building, with multiple taxa cooccurring in the same samples. Finally, even within-species diversity is also expected, as demonstrated by the cooccurrence of different *L. pneumophila* sequence types in the same water samples (Matthews et al. 2022).

Despite the demonstrated diversity, environment-specific *Legionella* spp. variability and the fundamental principles underlying cooccurrence of multiple *Legionella* species, sequence types, and their variants in the same environment remain largely unexplored. The high environmental diversity highlights a limitation in laboratory-research approaches, which typically focus on single strain experiments. Such approaches do not adequately represent the environmental dynamics given the considerable differences in the behavior/response of different *Legionella* species and strains. For example, several studies have observed considerable differences in temperature and protist–host preferences among different *L. pneumophila* strains and various non-*pneumophila Legionella* species (Ohno et al. 2008, Buse and Ashbolt 2011, Gomez-Valero et al. 2014, van der Kooij et al. 2016, Croze et al. 2021). Even growth on standard Buffered Charcoal Yeast Extract (BCYE) agar was distinct among different *Legionella* species (Lee et al. 1993). A deeper understanding of such (sub-)species-level differences would improve *Legionella* spp. management strategies by providing insights into how and why certain *Legionella* species and *L. pneumophila* strains proliferate in specific environments, and support the better alignment of clinical and environmental approaches (Proctor et al. 2022). Besides providing useful insights for *Legionella* spp. management, such understanding could inform the refinement of the media composition used for isolation (e.g. Descours et al. 2014) to aid *Legionella* spp. culturing and enable more accurate risk assessments, supporting the development of targeted control strategies.

Research opportunities and needs:

- There is a need for more extensive mapping of *Legionella* spp. diversity and cooccurrence in different environments, specifically linked to different sources and seasonality in their presence and concentrations (discussed below).
- There is a need to decipher the ecology of non*-pneumophila Legionella* species to better understand the conditions that drive their growth in engineered aquatic environments, and how that relates to the occurrence of *L. pneumophila* or other pathogenic species.
- There is an opportunity for the isolation and identification of the not-yet-cultivated *Legionella* species, which could be supported by a redesign of growth media and growth conditions based on available molecular data.
- There is the opportunity to pair genomic data, phenotypic characteristics, and occurrence patterns to identify critical genes responsible for observed environmental adaptation.
- There is an ongoing need to obtain growth preference, stress tolerance, and disinfectant susceptibility data among *Legionella* species and across different strains within a species, ideally under realistic yet reproducible and standardized environmental conditions, to better predict species and strain abundance and diversity in engineered ecosystems.

## All *Legionella* species are not (clinically) equal

Legislation often considers all *Legionella* species as equal, with many guidelines and management practices developed around *Legionella* spp. threshold values. As an example, the new EU Drinking Water Directive regulates a concentration of 1000 CFU/l *Legionella* spp. in drinking water (European Commission 2020). This is despite the facts that: (i) all current evidence suggest that *L. pneumophila* is responsible for the vast majority of waterborne cases, and (ii) that there exists no published dose–response data for any *Legionella* species other than *L. pneumophila* (Armstrong and Haas 2007) and *L. longbeachae* (Prasad et al. 2017). Focusing on all *Legionella* species potentially misappropriates critical time and financial resources toward remediation of systems with non-*pneumophila Legionella* posing minimal health risks. Here, we question whether risk-based legislation would be more sensible, supporting optimal resource allocation, and therefore, improved *Legionella* spp. management.

More than half of all *Legionella* species have been associated at least once with some type of clinical outcome (Cunha et al. 2016). However, numerous studies have shown that Legionnaires’ disease is predominantly caused by *L. pneumophila* (92%–99% of cases), with the remaining 1%–8% of culture-confirmed cases attributed to *L. anisa, L. bozemanae, L. dumofii, L. erythra, L. gormanii, L. longbeachae, L. micdadei, L. maceachernii, L. rubrilucens, L. turinensis, L. tucsonensis*, and *L. wadsworthii* (e.g. Yu et al. 2002, Doleans et al. 2004, Joseph et al. 2010, Svarrer and Uldum 2012, McMullen et al. 2024). Clear exceptions are Australia and New Zealand, where *L. longbeachae* is often the dominant disease-causing species (Chambers et al. 2021, Graham et al. 2023). Unlike other *Legionella* species, *L. longbeachae* is commonly associated with soil, compost, and potting mix rather than water systems. However, the causes for its higher clinical prevalence in Australia and New Zealand are still unclear. With respect to *L. pneumophila* dominance, detection/reporting bias is known (Kawasaki et al. 2022, Kim et al. 2022). For example, urinary antigen testing, which is the standard in clinical diagnostics, is designed to primarily detect only *L. pneumophila* serogroup 1, and a slight drop in the detection/reporting of other *Legionella* species was noted following the introduction thereof (Schoonmaker-Bopp et al. 2021). However, to our knowledge, no study outside Australia and New Zealand has ever reported high attribution (>10%) of culture-confirmed Legionnaires’ disease cases associated with *L*. non*-pneumophila* species. Moreover, multiple studies have demonstrated a disproportionate balance between the environmental prevalence of certain *Legionella* species and their clinical relevance (e.g. Doleans et al. 2004). This questions the practicality of focusing management and remediation resources on all *Legionella* species. In response to the new EU legislation, the Dutch government commissioned a report to assess the state of the knowledge and potential legislative changes. The main conclusion from this report was a recommendation to focus legislatively on *L. pneumophila*, and only use *Legionella* spp. threshold values in high-risk environments (van der Wielen et al. 2021). This mirrors similar legislative approaches in, for example, France which also focus on *L. pneumophila* opposed to *Legionella* spp. (Hartemann 2019). Using a large data set, Romano Spica et al. (2024) calculated the costs and benefits of focusing only on *L. pneumophila* opposed to *Legionella* spp. for routine monitoring, estimating this approach to have substantial economic and practical benefits with minor additional health risks. However, an approach targeting only/mostly *L. pneumophila* is not without a degree of risk and uncertainty (Delaney et al. 2022). First, a lesser-documented outcome of *Legionella* spp. infection is Pontiac fever, but this is underdiagnosed due to the relatively mild clinical presentation thereof (Edelstein and Roy 2015). Hence, the actual number of Pontiac Fever cases and the open question whether *L. pneumophila* is indeed the primary causative strain of Pontiac fever, remains unknown. Second, it is known that at-risk populations (i.e. immuno-compromised individuals) are more susceptible toward infection by non-*pneumophila* species (Muder and Yu 2002). Thus, based on current knowledge, it is sensible that environments with vulnerable people are legislated and managed to a higher degree (i.e. all *Legionella* species). Further justification for a more conservative focus on all *Legionella* species is the potential that such species can potentially be indicators for *L. pneumophila* presence (Crook et al. 2024), although there is currently no conclusive evidence for this. On the contrary, the absence of *L. pneumophila* detection in *Legionella* spp.-positive samples is common (van der Lugt et al. 2019). With respect to the latter, it is noted that some of the same best-practice management approaches to curb *L. pneumophila* proliferation are likely to deter other *Legionella* species as well. An alternative approach may be to target a group of the *Legionella* species associated with the highest disease burden during routine environmental monitoring, for example with a targeted multiplex PCR approach (Logan-Jackson et al. 2021). However, this would only be practical if there is a methodological switch toward replacing standard cultivation methods with quantitative PCR. Such a replacement is challenged by the fact that qPCR-measured concentrations often overestimate cultivation-based ones due to their sensitivity to DNA in nonviable cells and present extracellularly in the environment, as well as the influence of various factors and stresses on bacterial cultivability resulting, for example, in viable-but-not-culturable bacteria (Sylvestre et al. 2025). Moreover, such transition is not likely to find legislative acceptance soon. There is also a strong counter-argument to be made for the legislative prioritization of several other opportunistic microbial pathogens, notably *Pseudomonas aeruginosa*, nontuberculous mycobacteria, and *Aspergillus fumigatus* before the less relevant *Legionella* species are considered (van der Wielen et al. 2021).

Research opportunities and needs:

- Quantitative microbial risk assessment (QMRA) requires the generation of high-quality dose–response data for *Legionella* non-*pneumophila* species (and even for different *L. pneumophila* strains). To do this, suitable animal models or other approaches need to be developed and validated.
- There is a need for better interactions and collaborations with hospitals and clinical microbiology laboratories to support isolation and identification of causative species/strains of Legionnaires’ disease and Pontiac fever infections, including advancement of diagnostic techniques that are not biased toward *L. pneumophila* only (e.g. broader-range urinary antigen tests).
- There is a lack of data on coinfection by multiple species/strains and for QMRA dose–response relationship data for repeated infections.
- There is an opportunity to further develop, optimize, and apply multiplex quantitative PCR methods (including viability PCR methods) for clinically relevant species, improved PCR data interpretation, and better collection and sharing of such data from environmental samples.
- There is a need to collect detailed data sets recording the environmental prevalence of clinically relevant *Legionella* species, to better understand their contributions to disease burden, relative to their environmental abundance.
- There is a need for selected general population-based studies on the occurrence of various *Legionella* species, and also *Legionella* spp. antibodies, to understand prevalence in asymptomatic or mild infections.

## Different *Legionella* spp. sources are not equally important

**Figure 2.**
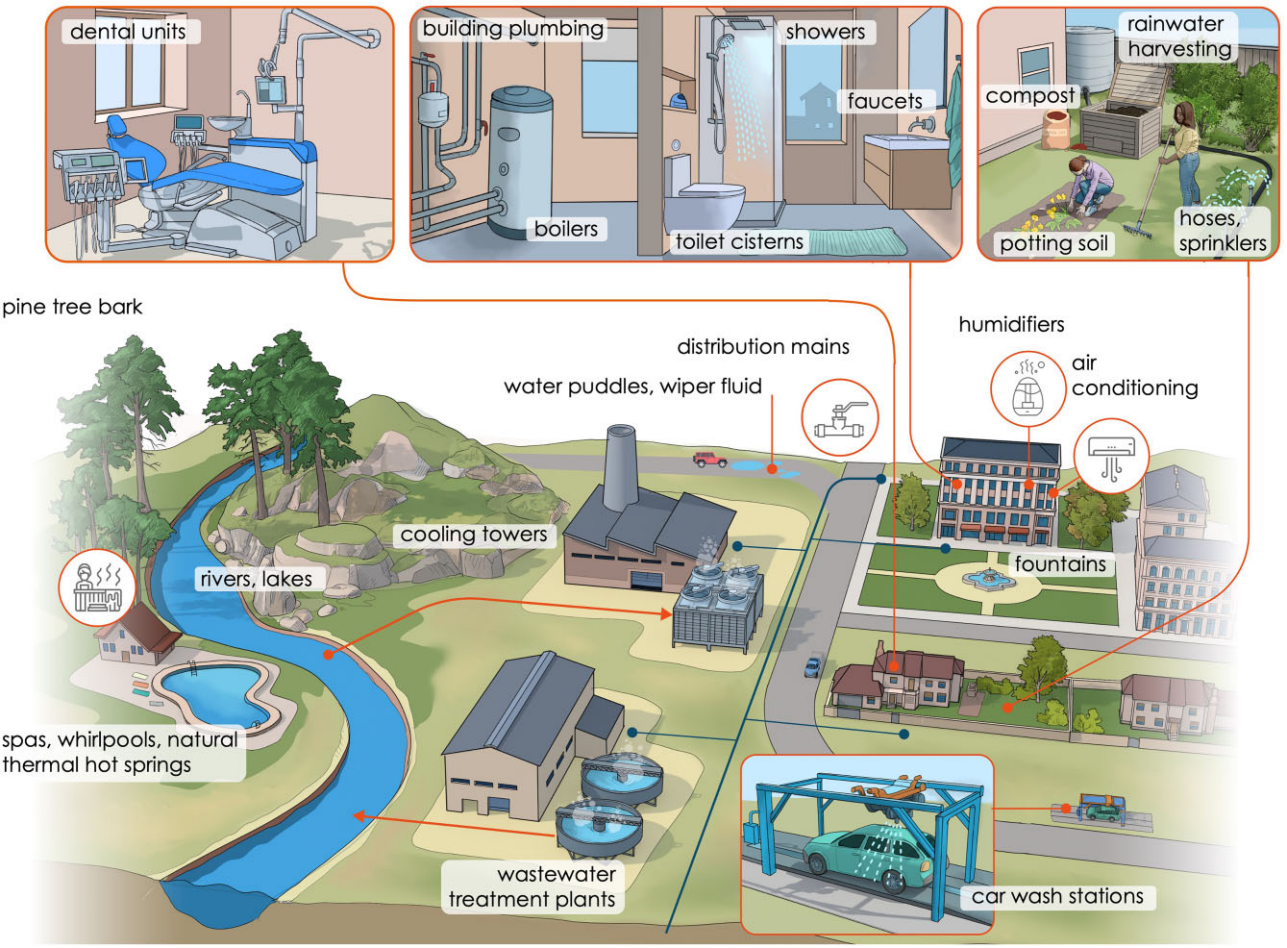
Examples of common sources of *Legionella* spp. in natural and engineered environments. Image: Rafael Pla Delgado.

Identification of the most significant sources of *Legionella spp*. responsible for clinically severe infections is important for case investigations (i.e. targeted sampling) and for policy making (i.e. best management practices). Members of the order *Legionellales* have been found at low relative abundance in most environments (Graells et al. 2018), likely thanks to the diversity of *Legionella* species (discussed above). A diverse range of infection sources have been identified previously (Fig. 2) (van Heijnsbergen et al. 2015, Prussin et al. 2017, Orkis et al. 2018). These include well-known sources such as cooling towers, spas, and building plumbing systems, but also potentially neglected sources such as wastewater treatment plants (Vermeulen et al. 2021), compost (Casati et al. 2010), and soil (van Heijnsbergen et al. 2014, 2016). Although it is reasonable to infer that we already know the main sources of *Legionella* spp., the source of infection for sporadic Legionnaires’ disease cases is usually not identified (Orkis et al. 2018), a fact that highlights the potential role of other sources. *Legionella* spp. have been isolated from a variety of unusual sources such as windshield water fluid (Wallensten et al. 2010), humidifiers (Moran-Gilad et al. 2012), heated birthing pools (Collins et al. 2016), surgical heating-cooling units (Ditommaso et al. 2020), and many others, which complicates the establishment of a comprehensive source list and hinders source attribution. Consequently, there is currently insufficient evidence to definitively rank sources in terms of their overall contribution to infections. While exposure frameworks have been developed for some indoor sources (Hamilton et al. 2019), a direct comparison of the potential risks posed by various sources has not yet been performed. Here, we considered the definition/classification of sources and the criteria to evaluate the importance of a source.

We distinguish between *proximate sources* (i.e. the point of dispersal), and *reservoirs* (i.e. the point of growth/accumulation, also sometimes referred to as “*ultimate sources*”), noting that these may be the same in some cases. For example, a defective hot water boiler in a building can be the reservoir of *Legionella* spp., but an individual shower unit would be identified as the proximate source where exposure occurs. This distinction is important because it affects how sources are managed. In the example above, construction (e.g. boiler type) and operation policies (e.g. temperature settings and disinfection) would affect the *Legionella* spp. reservoir, while shower head design and room ventilation could affect the proximate source. Proximate sources should be assessed based on (i) their ability and capacity to disperse *Legionella spp*. from the bulk water into the air, including the quantity of inhalable aerosols produced, (ii) the exposure of people to that source (magnitude of the exposure, number of people, and presence of at-risk people), and (iii) their connection to a reservoir. With respect to dispersal, *Legionella* spp. is dispersed in aerosols large enough to encapsulate the bacteria (>2 µm) and small enough to remain airborne and deposit within the alveoli of a person’s lungs (<5 µm) (Heyder et al. 1986). Hence, the ability to produce large quantities of aerosols in this range would elevate the importance of a source, such as the case of examples of showers, garden sprinklers, cooling towers, and wastewater aeration units. Multiple aspects related to exposure affect the importance of a proximate source. For indoor sources, this includes the size and ventilation of the space (affecting aerosol concentrations), and also the number of people exposed (Tang et al. 2025). For outdoor sources, the potential number of people exposed from a single proximate source may be much higher, and the environmental/weather conditions (Nguyen et al. 2006, Fischer et al. 2023b) and possibly the air quality (Yu et al. 2024) influence the aerosol dispersal. Naturally, the risk posed by a proximate source depends also on its context. For example, proximate sources in, or close to, high risk environments (e.g. hospitals and care facilities) should have a higher risk consideration.

*Legionella* spp. reservoirs are considered based on the degree to which the environment supports *Legionella* spp. proliferation in terms of both concentrations and species type/diversity. For example, a reservoir with moderate concentrations of *L. pneumophila* serogroup 1 most likely represents a higher risk than a reservoir with high concentrations of, for example, *L. antarctica*, a species for which no known cases have been reported. Linked to this, factors to consider are the availability of nutrients, optimal environmental conditions (e.g. temperature and pH), hydraulics (e.g. shear stress and stagnation), the presence of biofilms and thus the surrounding microbiome, and the presence of stress factors such as disinfectants. Many of these factors can also influence the thermo-physical properties of the water and thus impact the formation and dispersion of contaminated aerosol droplets. The management of engineered environments often dictates the degree to which they can be considered as reservoirs of *Legionella* spp.. For example, some studies suggested that water quality changes in distribution mains due to maintenance and/or operational decisions contributed to *Legionella* spp. proliferation and ultimately Legionnaires’ disease infections (Rhoads et al. 2017, 2020). Also, the hot water building plumbing system is not a source by default, but a poorly constructed or managed system can become a significant source of *Legionella* spp. growth. Recent studies of building closures during the COVID-19 pandemic highlighted how reduced occupancy and factors such as diminished water use and associated elevated water age may exacerbate the growth of *Legionella* spp. in colonized systems, where drinking water with a disinfectant residual is distributed (Hozalski et al. 2020, Grimard-Conea et al. 2022, Dowdell et al. 2023). Similarly, overgrowth may be a concern in buildings with oversized plumbing, as well as “green buildings,” which are characterized by increased hydraulic residence times and hot water temperatures in ranges more permissive for *Legionella* spp. proliferation due to their focus on water and energy efficiency (Yao et al. 2024). The resumption of water use or intentional use at high flow rates (i.e. flushing) may temporarily lower concentrations, but will not remediate underlying issues (Hozalski et al. 2020, Greenwald et al. 2022, Grimard-Conea et al. 2022, Grimard-Conea and Prévost 2023). Consequently, building codes and best management practices need to be implemented to protect such systems from becoming *Legionella* spp. reservoirs. Previous studies report *Legionella* spp. concentrations and/or species for different sources (e.g. Prussin et al. 2017, Salinas et al. 2021), while others documented detailed data for a specific type of environment such as potable/drinking water (Dilger et al. 2018, van der Lugt et al. 2019). However, the broad range of *Legionella* spp. sources (Fig. 2) are often investigated only from an infection perspective, but are not necessarily well-documented from a broad environmental microbiology perspective. This currently limits our understanding of the causes leading to the establishment of a *Legionella* spp. reservoir.

Using QMRA, risk-based concentration targets can be defined for *L. pneumophila* for various water sources based on quantitative relationships with health outcomes (Hamilton et al. 2019). Key QMRA input parameters, such as exposure duration, inhalation rate, population susceptibility, and dose–response choice, directly influence the resulting concentration targets (Tang et al. 2024). These concentration targets enable the evaluation of monitoring results against health-based standards, such as a risk level of 1 × 10^−6^ disability-adjusted life years per person per year. However, to assess compliance with these concentration targets, it is essential to compare them to the distribution of the concentration over the exposure period relevant to the health-based standard, typically 1 year. Rather than relying only on a point estimate, establishing a confidence interval for this average concentration helps account for statistical uncertainty, ensuring that the mean concentration accurately reflects the true exposure level in relation to the health-based standard. Therefore, the implementation of risk-based concentration targets could greatly benefit from close integration with statistically valid monitoring strategies. However, putting such strategies into practice for *Legionella* spp. in various sources is challenging because *Legionella* spp. concentrations can increase rapidly—by up to 2.0-log in just a few weeks in case of suitable conditions for growth (Lee et al. 2011a, Völker et al. 2016). Such fluctuations can easily be missed, even with relatively frequent monitoring (e.g. monthly). Furthermore, spatially localized *Legionella* spp. growth in complex engineered systems, such as hospital plumbing (Jeanvoine et al. 2023) and cooling towers (Trigui et al. 2024), complicates the collection of representative samples necessary for reliable statistical analyses, as such growth can be missed with limited routine samples. Consequently, while the low-frequency monitoring and targeted sampling strategies currently in use may provide insights into the efficacy of control measures, they offer only a limited understanding of *Legionella* spp. concentration distributions and subsequent risk. This limitation hinders the ability to assess compliance with the health-based standard. To overcome these challenges, an alternative or complementary approach is to develop predictive microbiology models—which were originally developed in food microbiology—that predict changes in the numbers (or concentrations) of pathogens under various environmental conditions (Mataragas et al. 2006). Developing predictive *Legionella* spp. growth models that integrate environmental variables and microbial ecology factors could reduce uncertainties associated with *Legionella* spp. concentrations and facilitate more targeted interventions.

Research opportunities and needs:

- There is a need for the characterization of the aerosol generation mechanisms and the transfer of *Legionella* spp. from the bulk water to aerosols in order to estimate the risk from proximate sources accurately.
- There is an opportunity to develop and implement evidence-based QMRA models, potentially incorporating predictive growth modeling, to help identify and rank critical sources for both routine monitoring and case investigations.
- There is a clear need for broad quantitative and qualitative data on *Legionella* spp. diversity and abundance in the environment, using alternative quantitative and advanced sequencing approaches, to enhance understanding of temporal and spatial fluctuations in environmental reservoirs.
- There is a need for predicting alternative not-yet identified sources, such as sources that become relevant through climate change or new equipment (e.g. misters, pressure washing equipment, LED-mist fireplaces, and evaporative coolers).
- There is an opportunity to identify the characteristics and adaptations required by distinct *Legionella* species for their survival and establishment in specific environments.
- There is a need to better quantify the factors that impact *Legionella* spp. movement and survivability through a macro-level environment (e.g. allowing for several kilometers of travel through the air).
- There is a need for interdisciplinary collaboration between scientists, industry, and legislative agencies dealing with the diverse factors and environments that constitute a source (e.g. plumbing systems, water quality, aerosol formation, air quality, exposure, and so on).

## What drives seasonality in Legionnaires’ disease cases?

**Figure 3.**
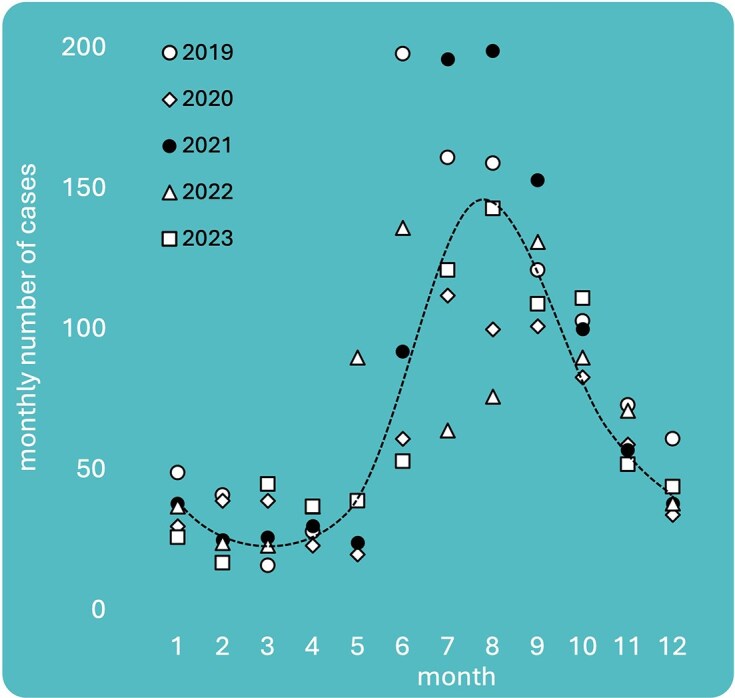
Example of seasonality in Legionnaires’ disease cases in New York State (2019–2023), with often 3–5 times more cases in summer than in winter. Data: New York State Department of Health. Month 1–12 is January–December, with 6–9 depicting the Northern Hemisphere summer months.

Legionnaires’ disease cases are seasonal, with the incidence rates in summer often 3–5-fold higher than in winter (e.g. Fisman et al. 2005, Alarcon Falconi et al. 2018). For example, seasonality is consistently observed in all age groups, as well as genders and geographic location, being independent of varying regional notification rates over decades (Fischer et al. 2020). Figure 3 shows seasonal case data from the USA in New York state for the last 5 years, and similar data have been recorded for many countries, albeit with clear regional differences. Multiple epidemiological studies investigated Legionnaires’ disease seasonality, and correlated incidence rates to meteorological variables, predominantly temperature, humidity, and rainfall (e.g. Karagiannis et al. 2009, Simmering et al. 2017, Fischer et al. 2023b). To parse this out further, Fischer et al. (2023b) applied a detailed modeling approach to Swiss data, showing that more cases occur specifically 6–12 days following warm weather and 6–14 days following increases in relative humidity. The importance of such factors, as well as their combination, has also been noted in different geographical settings, suggesting the general validity of this association (Brandsema et al. 2014, Simmering et al. 2017). Given that these data refer only to the number of reported cases, the information pertaining to the mechanism(s) linking these (and other) seasonal factors to *Legionella* spp. presence in the environment, dispersal, and eventual infection of humans is still largely lacking. A mechanistic understanding of seasonality may provide opportunities to better manage infection risks during these critical periods. Here, we opine on various seasonally related factors, including meteorological patterns, human actions/activities, and system operations, and how these are potentially interlinked with *Legionella* spp. growth, dispersal and exposure.

The risk of a *Legionella* spp. infection theoretically increases due to any of the following factors: (i) when the *Legionella* spp. concentrations in/at a given reservoir increase (e.g. through growth); (ii) when *Legionella* spp. infectivity and/or virulence increases in a given source [e.g. a community shift toward more pathogenic (subspecies)]; (iii) when the number of sources increases; (iv) when dispersal from a single source increases; (v) when survival in aerosols increases (meaning increased dispersal and, thus, also likely exposure); (vi) when exposure to sources or aerosols increases through human activities/actions; and (vii) when susceptibility to infection in humans increases. We explore from a source perspective the impact of seasonality on these factors with two examples. Our first example is the use of cooling towers, a commonly recognized source of *Legionella* spp. infections (Fitzhenry et al. 2017). Particularly small and medium size towers represent at the same time reservoirs and proximate sources and are either used more intensively or uniquely in summer (i.e. increased number of sources or increased dispersal from single source). Warmer weather means growth conditions in the towers change, potentially leading to higher concentrations of *Legionella* spp. and potential shifts in community (i.e. different *Legionella* species) (Di Gregorio et al. 2018, Tsao et al. 2019). In case of increased humidity, *Legionella*-containing aerosols are, then, likely to evaporate slower and thus disperse further. Due to human activity/actions that are increased in the warmer months (e.g. walking outside and keeping windows open), individual exposure to the aerosols would increase. Thus it is, at least theoretically, possible to identify multiple seasonal exacerbating factors. The second example is the activity of gardening, which has been previously linked to *Legionella* spp. infections (Den Boer et al. 2007). Gardening is clearly a seasonal activity, often practiced by older people (i.e. increased general population exposure and potentially an increase in high-risk individuals exposed in summer). It typically involves direct interaction with known *Legionella* spp. reservoirs including soil and compost (Casati et al. 2010, van Heijnsbergen et al. 2014, 2016), garden hoses (Thomas et al. 2014), and sometimes collected rainwater (Dobrowsky et al. 2014, 2017), thus increasing the number of *Legionella* spp. sources to which the person is exposed. In the case of garden hoses and collected rainwater, the warm and stagnant conditions would furthermore likely lead to *Legionella* spp. proliferation and potential community shifts, although this has not been investigated in detail (Thomas et al. 2014). Again, it is possible to identify multiple seasonal exacerbating factors. These illustrative examples highlight the potential multifaceted nature of seasonality in *Legionella* spp. infections. Similar examples can be generated for various sources and activities, but actual data or specific models to quantify these seasonal changes are lacking. Nonetheless, while no single specific source or activity has been linked decisively to the seasonality of *Legionella* spp. infections, most of the factors associated with seasonality point strongly toward outside environments/sources, suggesting that primary transmission routes in summer may not necessarily be through building plumbing systems. A counterexample to this would be *Legionella* spp. proliferation in drinking water distribution networks during summer. There are some networks where seasonal temperatures increase well within the range of *Legionella* spp. growth (e.g. Sharaby et al. 2019). This would be more relevant in regions experiencing climates warm enough to result in a sufficient increase of drinking water temperature. While, to our knowledge, current epidemiological data do not support this as the main infection route in summer, growth in the network would contribute to seeding *Legionella* spp. reservoirs, and thus increase the probability of *Legionella* spp. growing there. It would nonetheless be interesting to model whether a relatively small increase in *Legionella* spp. in the distribution network would account for a sufficiently increased number of cases in summer given the large population affected. Based on the current evidence, we conclude that many factors combine to give seasonal data, and these may not even be the same in all regions/countries and that prevention/management strategies are likely to be adapted depending on local conditions.

Finally, it is worth highlighting that *Legionella* spp. seasonality might be affected by the change in seasons’ duration and intensity caused by climate change (Wang et al. 2021), as well as the frequency of extreme weather events (Clarke et al. 2022). In fact, given the link between temperatures and humidity highlighted earlier, it is expected that Legionnaires’ disease cases will keep increasing due to the effect of climate change (Walker 2018, Han 2021, Dupke et al. 2023). Furthermore, warmer conditions will potentially increase the use of known *Legionella* spp. sources such as cooling towers and air conditioning units (Hamilton et al. 2018), as well as increased engagement to activities linked to exposure, such as gardening (Bisgrove and Hadley 2002). Beyond meteorological characteristics and human behavior, increased temperatures will also affect the biology of *Legionella* spp., as well as their interactions with other microorganisms. First of all, higher temperatures will likely accelerate environmental growth rates of *Legionella* spp. (Sharaby et al. 2017). With respect to water supply systems, several European countries such as Switzerland distribute tap water without residual disinfectants. Accordingly, such distribution networks are unprotected against the proliferation of *Legionella* spp. when the temperature rises (e.g. in summer and/or due to climate change). The sporadic detection of *L. pneumophila* in water supplies increases with temperature higher than 25°C (van der Kooij and van der Wielen 2013), which may well be reached in more-and-more distribution systems during the summer in the next few years with climate change. Furthermore, it is known that certain *Legionella* spp. hosts, such as *Naegleria fowleri*, are thermophiles and their presence is expected to increase with the presence of warmer waters (Heilmann et al. 2024), potentially resulting in more hosts available for *Legionella spp*. replication. In addition, it has been observed how temperatures below 20°C allowed amoebae to digest intracellular *L. pneumophila* and *L. micdadei*, in contrast with the replication observed at higher temperatures (Ohno et al. 2008, Gomez-Valero et al. 2014). Such a result suggests that increased periods with warmer temperatures might also allow for more replicative niches, further increasing *Legionella* spp. concentrations, as well as potentially shifting *Legionella* spp. diversity, in the environment.

Research opportunities and needs:

- There is an opportunity to comparatively analyse existing seasonal case data from different regions, particularly where seasonality (and/or the factors related to *Legionella* spp. infections) is more pronounced. This, however, comes with the caveat that high quality data and metadata are not always available.
- There is a clear need for quantitative data on seasonal fluctuations in *Legionella* spp. in terms of both concentrations and species in critical sources, as well as a mechanistic understanding of how environmental changes drive these fluctuations.
- There is a need for careful consideration of how climate change may affect seasons, weather patterns and extreme events (e.g. longer warm periods and more rainfall events) on both large and local scales. The effect of these changes on *Legionella* spp. growth and transmission and by inference Legionnaires’ disease seasonality should then be estimated, assessing also whether mitigation strategies against increased risk can be developed.

## Hot water temperatures challenge energy-saving requirements in buildings

Building plumbing systems are a known source of *Legionella* spp., and failing to maintain adequate hot water temperatures is recognized as one reason for unwanted *Legionella* spp. colonization and growth. A generally agreed rule-of-thumb is that hot water storage tank temperatures exceeding 60°C (140°F) is mostly sufficient to control *Legionella* spp. presence in circulation systems, even when considering the inevitable heat losses during recirculation. The challenge is that conventional hot-water heating in buildings consumes up to 17%–39% of a building’s energy costs (Armstrong et al. 2014, Völker and Kistemann 2015, Booysen et al. 2019, Pomianowski et al. 2020), while not necessarily guaranteeing the complete absence of *Legionella* spp.. Here, we consider the options and trade-offs when lowering hot water temperatures to conserve energy, whether/how that affects *Legionella* spp. growth in the final meters, and specifically whether additional on-site chemical disinfection presents a reasonable and sustainable means to achieve lower energy consumption in building water-heating systems.

**Figure 4.**
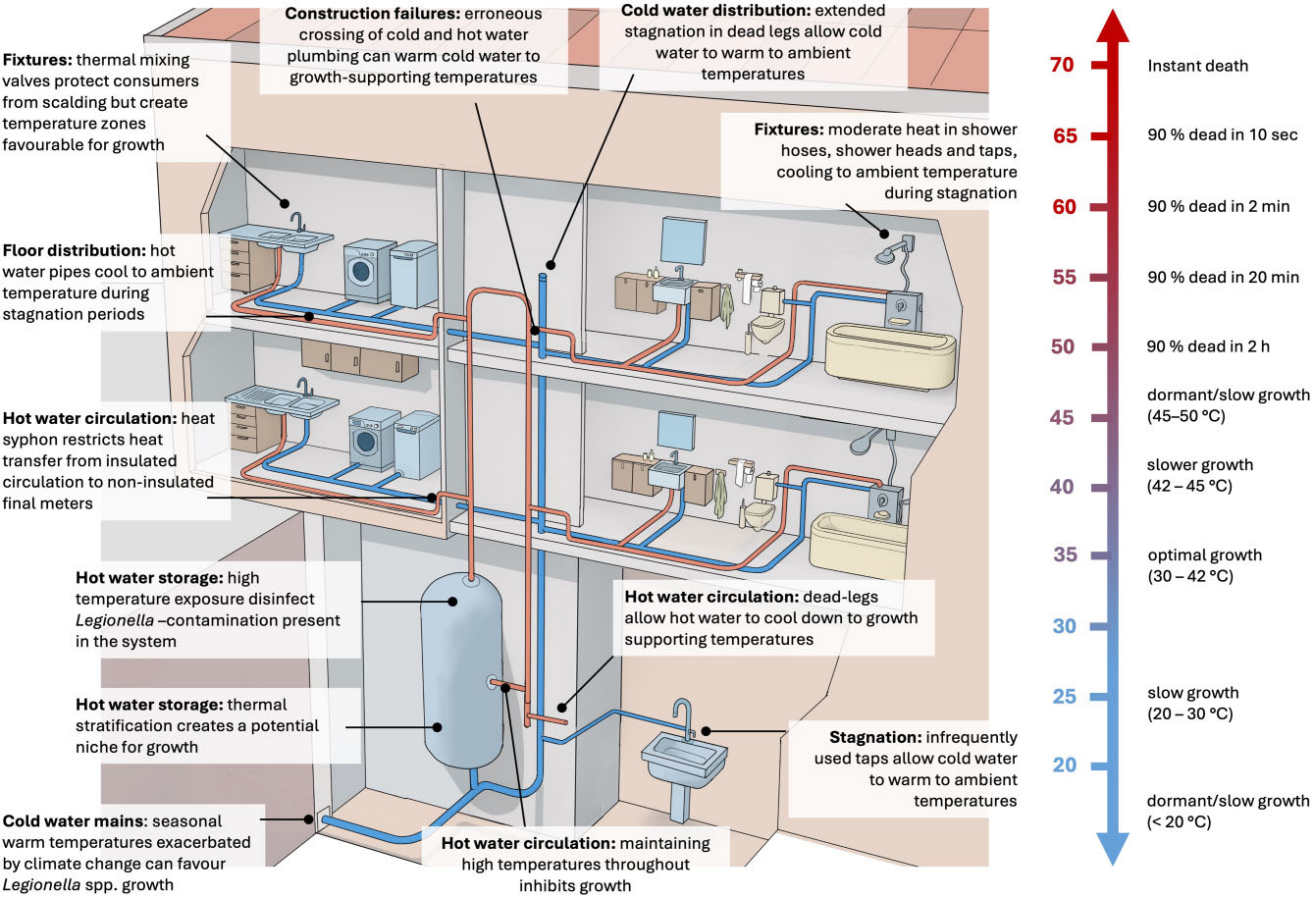
Temperature fluctuations in the potable water supply of a building and the impacts thereof on *Legionella* spp. growth, survival, and death. The temperature responses of *Legionella* spp. are generalized; species-specific differences can occur. Image: Rafael Pla Delgado.

In simplified terms, a building plumbing potable water system comprises a hot water storage tank and circulation/distribution system, and a cold-water distribution system (Fig. 4). From a temperature perspective this is more complicated, with for example heat loss during hot water distribution, and heat gain during cold water distribution, respectively. The broad range of temperatures (and temperature fluctuations) that can occur in a building mean that without optimal design and operation, niches for diverse *Legionella* species can develop. From a hygienic perspective the underlying principle of *Legionella* spp. control is thus to keep the building plumbing system either hot enough or cold enough to limit *Legionella* spp. growth. In this context, the primary purpose of a building’s hot water system is to prevent *Legionella* spp. from establishing and proliferating in the system in the first place, while a secondary purpose is to disinfect any *Legionella* spp. that may be present. The first point to consider is the theoretical hot water temperature requirements to control *Legionella* spp.. Many *Legionella* species are thermotolerant, and survive extended exposure to temperatures above 50°C (Cervero-Aragó et al. 2015, Papagianeli et al. 2021), with some species or strains potentially surviving or adapting to even higher temperatures, especially in case of repeated heat exposure (Allegra et al. 2011, Liang et al. 2023). However, *Legionella* spp. grows optimally below 42°C and to our knowledge, no study to date has conclusively demonstrated *Legionella* spp. growth above 50°C (121°F) on laboratory scale in culture media (Kusnetsov et al. 1996, Hochstrasser and Hilbi 2022). This latter point is challenged by multiple field sampling campaigns from real buildings that detected viable *Legionella* spp. in plumbing systems above 50°C (Rasheduzzaman et al. 2020, Grimard-Conea et al. 2024, Kistemann et al. 2024). The likely explanation is the failure of complex buildings to maintain high water temperatures throughout the entire system (e.g. due to dead legs, hydraulic failures, or stratified hot water storage tanks) (Kistemann et al. 2024). Hence, a first step toward satisfying both energy saving and hygienic safety is to minimize heat losses and hydraulics across the entire hot water system to maintain control temperatures in the return circulation loops and as much as feasible at the periphery near the points of use (Bédard et al. 2015, Kistemann et al. 2024). This can only be achieved if hydraulic deficiencies are addressed (Blanc et al. 2005, Boppe et al. 2016). Indeed, most regulations and guidance on thermal regimes require either minimum return loop temperatures or maintaining minimum temperatures across the whole system. In this respect, 55°C is typically viewed as the target temperature for the insulated hot water circulation system (Rasheduzzaman et al. 2020), while Kistemann et al. (2024) concluded from a particularly large data set (>290 000 samples) that the tipping point where problematic *Legionella spp*. growth is observed is 53°C (127°F). Evidently, there is only limited flexibility to save energy through temperature reductions alone, but even small decreases in hot water setpoints can result in substantial energy savings. However, the optimal operating temperatures are building-specific and decreasing building hot water temperatures require detailed temperature and microbial monitoring to ensure safety of the consumers. Importantly, temperature in the system and in the circulation return loops may not be predictive of positivity in peripheral samples as many other factors such as stagnation, heat-loss, materials and surface to volume ratio come into play. The final (noncirculating) meters of a plumbing system are only exposed to hot water intermittently and for short durations, with stagnation and cool-down to ambient temperature the norm. It is well known that these distal sections are more often colonized by *Legionella* spp., irrespective of circulation temperature (Kistemann et al. 2024). Moreover, the presence of *Legionella* spp. in some distal sections is attributed to growth in cold water plumbing sections (Wüllings and van der Kooij 2006, van der Lugt et al. 2019), which are obviously not influenced or managed by hot water settings. Ultimately, temperature control alone may not always suffice to control *Legionella* spp. in buildings and other control measures may need to be introduced.

Additional on-site chemical disinfection is a possibility for new and legacy buildings with complex and/or old plumbing systems, particularly where high risk consumers are involved [e.g. hospitals, (health)care facilities, long-term care homes, and so on]. Several on-site disinfection technologies have been applied to control *Legionella* spp. in building water systems including free chlorine, chloramines, chlorine dioxide, and copper–silver ionization. Considering 20 field studies, Xi et al. (2024) concluded that the relative efficacy of chlorine-based disinfectants to control *Legionella* spp. in building water systems was in the order of chloramine > chlorine dioxide > chlorine. In recent years, monochloramine (NH_2_Cl) has increasingly been used for building-scale disinfection due to the relatively low concentrations of characterized disinfection byproducts produced as well as its stability, allowing for more effective disinfection in distal parts of the water system and deeper penetration into biofilms (Lee et al. 2011b). Monochloramine has been shown to be highly effective to rapidly decrease prevalence and even eradicate *Legionella* spp. and *L. pneumophila*, more effectively than free chlorine (Marchesi et al. 2012, Wang et al. 2013, Casini et al. 2014, Duda et al. 2014, Mancini et al. 2015, Coniglio et al. 2018, Lytle et al. 2021, Dowdell et al. 2023). Its usage has been associated with lower frequencies of detection of environmental *Legionella* spp. and their hosts and disease outbreaks (Kool et al. 1999, Flannery et al. 2006). Notwithstanding these apparent advantages, it is essential that any in-building disinfection approach considers the building attributes (e.g. pipe material) and particularly the end-use to avoid any unintended consequences. Apart from providing an additional hygienic barrier to *Legionella* spp., this approach is also viewed by some as an option that could permit to lower water temperatures and safely meet energy conservations goals. Relying solely on on-site disinfection without maintaining elevated hot water temperatures has been documented through some extensive field studies. One study in a healthcare clinic showed that a small decrease from 60°C to 57°C increased the prevalence and standard exceedances for *Legionella* spp. except when chlorine dioxide disinfection was present (Völker and Kistemann 2015). A separate 3-year longitudinal study conducted at an 870-bed tertiary care hospital documented the efficacy of on-site disinfection (ozonation and copper–silver ionization) after lowering the hot water setpoint to 50°C for energy conservation (Blanc et al. 2005). In that study, both water and biofilm swabs showed elevated positivity in both the ozone-treated networks (65%–56%) and in copper–silver-treated systems (90%–93%), suggesting challenges when lowering temperatures in complex buildings. On the other hand, an extensive case-study on a new hospital building in the UK suggests that careful commissioning and operation from the start at low temperature (42°C–43°C), but with copper–silver treatment for *Legionella* spp. control can be effective (Cloutman-Green et al. 2019). The study assessed 1600 samples during 6 years and reported no *Legionella* spp. positive sample by culture, with estimated energy savings of 33% compared to an equivalent temperature-controlled system. The data are encouraging, but it is obviously limited to a single building, operated from the start with chemical disinfection and extensive flushing at all points of use.

The benefits of on-site disinfection should be evaluated considering the trade-offs of different disinfectant types against broader health risks such as disinfection byproducts, selective enrichment of unwanted microbes, and mobilization of heavy metals (Gagnon et al. 2004, Waak et al. 2019, Quon et al. 2024). Elevated concentrations of oxidative disinfectants can accelerate the aging of plumbing materials (Rockaway et al. 2007, Castillo Montes et al. 2012, 2014). This should be factored into costs and overall energy savings. Onsite disinfection, particularly when considered as an option to lower water temperatures below recommended set-points, requires additional monitoring of residual disinfectant as well as *Legionella* spp. to ensure chemical and microbiological standards are maintained throughout a building’s plumbing system. While chemical disinfection may save energy on-site by allowing for lower temperatures, its installation and operation still requires energy and chemicals whose environmental footprint should be taken into consideration. Hence, we conclude that while (additional) chemical disinfection may well be required to control *Legionella* spp. in some buildings, its use should complement thermal control rather than substitute it. Any permanent reductions in thermal control below recommended temperatures should be approached with caution and only implemented with high-frequency monitoring programs, comprehensive system evaluations, expert supervision, and consideration of health risks to users, supported by comprehensive decision-making supporting information for building managers and end-users alike (Völker and Kistemann 2015, Roy et al. 2021).

Finally, for both thermal and chemical disinfection to be effective, it must act on all sources of *Legionella* spp. in a building system. It is worth noting that the vast majority of tests regarding *Legionella* spp. susceptibility to temperature and disinfection in the laboratory and in the field have been performed on water considering the bacteria as free-living. However, biofilms are the most important microbial reservoirs that can be persistent sources of *Legionella* spp. and their hosts, contaminating the bulk water (Healy et al. 2024, Silva et al. 2024). At any given time, a considerable fraction of the *Legionella* cells present in a plumbing system will be internalized in their protist hosts. The limited research on the topic has shown how inactivation kinetics of both chemical and thermal inactivation are largely reduced for host-associated *Legionella* spp., even though the extent of this can vary even among different strains of the same host (Cervero-Aragó et al. 2015). In addition, the presence of hosts can allow *Legionella* spp. concentrations to recover after heat treatment (Cazals et al. 2022), providing a further mechanism for *Legionella* spp. proliferation in plumbing systems in case of only partial inactivation.

Research opportunities and needs:

- There is the need for further optimization of engineered water systems (e.g. boilers and building plumbing systems) to avoid creating conditions where *Legionella* spp. can establish and grow, as well as developing strategies to retrofit existing systems.
- There is a clear data gap to determine whether the growth of *L. pneumophila* and *Legionella* spp. in bulk and biofilm can be controlled in hot water systems operated at lower temperature setpoints to meet energy conservation goals, if operated with effective onsite disinfection. This is especially lacking in legacy building water systems.
- The development of better online/inline monitoring (temperature and flow) systems for buildings offers the opportunity for developing more dynamic and efficient building management strategies.
- There is a need to develop clear and transparent communication strategies between building managers and building residents regarding available data and potential risks.
- There is a need for quantification of the economic, environmental and exposure-risk trade-offs of implementing additional/on-site chemical disinfection in buildings (e.g. system-scale life cycle analysis of the various alternatives).
- In order to design proper management strategies, it is necessary to better understand the role of protist hosts in *Legionella* spp. resistance to environmental stresses.

## Amongst others: the importance of *Legionella*-associated microbiome research

The microbiomes of engineered aquatic systems are complex, dynamic, and variable based on regional, temporal, and operational differences (e.g. source water, treatment methods, disinfection practices, and temperature) (Di Gregorio et al. 2017, Zhang et al. 2017, Paranjape et al. 2020b, Palanisamy et al. 2022). In many engineered aquatic environments, different *Legionella* species are natural members of the resident microbial communities, with the potential to interact positively and negatively with other microorganisms across trophic levels. It is generally agreed that *Legionella* spp. survives as free-living bacteria in biofilms, but proliferates through protists (Declerck 2010, Mondino et al. 2020, Barbosa et al. 2024). However, the extent of these environmental dynamics is not yet fully elucidated such as, for example, the ratio of free-living to host-associated *Legionella* cells at any given moment in a system. Here, we opine on why knowledge of *Legionella–*microbiome interactions is valuable for improved system design, operation, and management to minimize conditions favoring *Legionella* spp., and support the development of potential microbiome-based control and/or environmental strategies against *Legionella* spp. proliferation in engineered environments.

**Figure 5.**
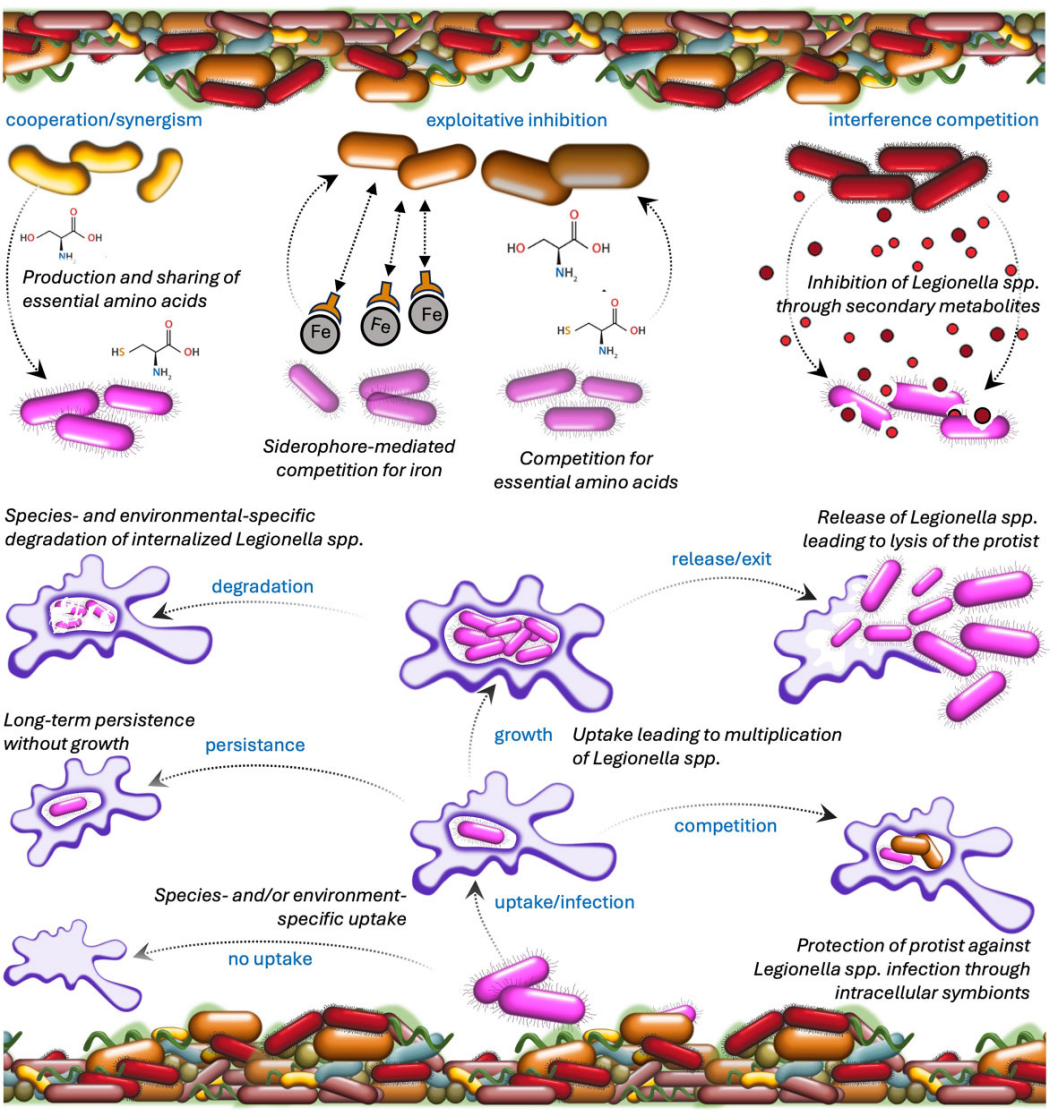
Possible interactions between *Legionella* spp. and other prokaryotic and eukaryotic members of the microbial communities in the polymicrobial biofilms of natural and engineered aquatic environments.

Several bacterial species are capable of directly inhibiting free-living *Legionella* species through the production of antimicrobial compounds such as various biosurfactants (Loiseau et al. 2015, 2018, Cavallaro et al. 2024), warnericin RK (Verdon et al. 2008), and toxoflavin (Faucher et al. 2022) (Fig. 5). However, the inhibition of *Legionella* spp. with these compounds has never been demonstrated to occur in real environmental settings. Moreover, because *Legionella* species are auxotrophic for some critical amino acids (Ewann and Hoffman 2006) and require iron for growth and infection (James et al. 1995), indirect competition for nutrients from bacteria that scavenge amino acids and iron is also plausible (Cavallaro et al. 2023, Vollenweider et al. 2024). However, these nutrient requirements also have the potential to support synergistic/cooperative interactions, for example with *Legionella* spp. growing in concert with bacteria that overproduce and/or leak amino acids (Paranjape et al. 2020a, McKinlay 2023). Early experiments demonstrated, for instance, that the presence of other microorganisms can promote the growth of *Legionella* spp. on agar plates not supplemented with key nutrients (Tison et al. 1980, Wadowsky and Yee 1983, Toze et al. 1990). However, these mechanisms have not been explored sufficiently to date, especially with respect to the host-associated *Legionella* spp. state. In fact, once internalized, *Legionella* spp. can exploit protists’ metabolism to recover required nutrients without competition of other extracellular microorganisms, overcoming competition for resources as highlighted above. Still the hosts’ cytoplasm is yet another environment, where microbial competition and potentially cooperation can occur. For example, intracellular *Parachlamydiaceae* bacteria have been shown to prevent *L. pneumophila* replication within hosts by either preventing its entry (Maita et al. 2018) or by hampering the development of its transmissive form through nutrient competition (König et al. 2019). In addition, it was shown that certain anti-*Legionella* spp. molecules, such as surfactin, show some activity against amoebae (Loiseau et al. 2015) and that specifically biosurfactants are able to disrupt biofilms (Bonnichsen et al. 2015), thus affecting the reproductive niches and environmental reservoirs of *Legionella* spp. that are difficult to target with standard mitigation strategies.

Beyond laboratory experiments, the presence of *Legionella* spp. has been associated with high cell numbers, suggesting that the thriving microbial communities can support the opportunistic pathogen (Proctor et al. 2018). In fact, several studies have investigated the *Legionella*-associated microbiome to elucidate this, finding both negative and positive statistical relationships with other bacteria (Ma et al. 2020, Paranjape et al. 2020b, Scaturro et al. 2023, Kanatani et al. 2024). While all these studies have identified taxa related to the presence/absence of *Legionella* spp. in specific samples, it seems evident that a general *Legionella*-associated microbiome does not (yet) materialize from the combined data. There are several plausible explanations for this. For example, *Legionella* species are diverse, and most studies in this field employ methods that do not allow for the detection of organisms at the species level (e.g. Cavallaro et al. (2023)). The associations are then analysed and generalized at higher taxonomic levels (e.g. genus), providing results which are confounded by the heterogeneity within these taxonomic levels (i.e. different relative abundance of the species within a genus across samples) and lack finer resolution. Also, it is common knowledge that taxonomic identity does not necessarily infer functionality (Louca et al. 2016), and it is therefore possible that taxonomically distinct microbiomes have similar interactions with *Legionella* spp. that are not captured with amplicon sequencing. Furthermore, the presence of high-order interactions (e.g. interaction of more than two microorganisms, either prokaryotic or eukaryotic), as well as their context-dependency, hampers the retrieval of meaningful interactions from amplicon sequencing data alone, as well as their simulation in laboratory settings (Ludington 2022). For example, it is possible that a certain bacterial species might not significantly affect the abundance of *Legionella* spp. in an environment (i.e. no statistical association), but that this species could act as prey for one of several *Legionella* spp. hosts, offering as a result greater opportunities for *Legionella* spp. replication (i.e. positive statistical association) (Paranjape et al. 2020a). Similarly, the presence of an endosymbiont protecting a potential host can potentially shift a positive association between the host and *Legionella* spp. to a nonsignificant one (König et al. 2019).

The potential interactions between *Legionella* species and protists in the environment are even more complex (Fig. 5). The most-described interaction is that *Legionella* spp. are phagocytized by protists (e.g. amoebae), establish in a so-called *Legionella*-containing-vacuole, and then hijack the degradation mechanism of the protist to proliferate and eventually exit the cells often leading to the demise of the latter (Shaheen and Ashbolt 2021). However, a wide variety of alternative outcomes are possible, depending on the protist host, the *Legionella* species, and the environmental conditions. For example, *Legionella* spp. are in some cases not able to escape the host’s degradation mechanisms and are hence digested (Boamah et al. 2017), or different *Legionella* species and strains respond differently to the exact same protist host (Buse and Ashbolt 2011). The outcome depends primarily on the repertoire of Icm/Dot effectors carried by *Legionella* spp. to take control of the protists (Gomez-Valero et al. 2019) and on the bacteria life cycle (Molofsky and Swanson 2004). Importantly, *Legionella* spp. resistance to protist phagocytosis leads to enhanced resistance to phagocytosis by immune cells such as macrophages since they share similar mechanisms (Escoll et al. 2013). Again, these interactions have mostly been observed in laboratory-based experiments. A knowledge gap exists with respect to the environmental outcomes of these mechanisms, and how host specificity may affect fitness within specific engineered environments (Ensminger et al. 2012). Although more and more studies characterize *Legionella*-associated protists (Vilne et al. 2021, Cavallaro et al. 2023), these tend to suffer from the fact that protists in general are poorly characterized, and both the methodologies (i.e. 18S rRNA sequencing) and the databases for protist identification still lag well behind that of bacteria (Gao et al. 2024). In fact, while it is known that *Legionella* spp. have broad host ranges (Thomas et al. 2010, Boamah et al. 2017, Nisar et al. 2020), very little information is present about its extent and how these change between species and strains. One further layer of complexity is due to the fact that the outcome of *Legionella*-protists interactions can be influenced by environmental factors (e.g. temperature; Ohno et al. 2008) and also potentially by the presence of other microbiome members (e.g. Maita et al. 2018, König et al. 2019). Moreover, in low density environments, protists have been shown to have predation preferences and to predate on *Legionella* spp. as a last resort (Shaheen and Ashbolt 2021); however, there is no data regarding the persistence and impact of such preferences in complex biofilms. These considerations show both the relevance of protists for *Legionella* spp. persistence in the environment, as well as the critical shortcoming of the methodologies to investigate protists in the environment and the fundamental knowledge gaps in their ecology.

Research opportunities and needs:

- There is the opportunity to establish standard methodologies (e.g. sequencing and analyses) for both prokaryotes and eukaryotes to compare results across studies and leverage recent advances in machine learning.
- There is the need to move beyond a pure taxonomy-based approach and consider the functional potential of microorganisms to understand the microbial interactions which support *Legionella* spp. establishment in microbiomes.
- A clearer understanding of the environmental niche, the host range, and the ratio between free-living to host-associated cells of each *Legionella* species is required to elucidate *Legionella* spp. distribution in the environment.
- There is an opportunity to develop microbiome-based control strategies by understanding the trophic and ecological interactions between individual *Legionella* species, (host) protozoan community and the (prey) bacterial community in water systems. The establishment of such control strategies will require validating *Legionella* spp. inhibition from compounds produced by other microbes in realistic conditions.
- There is an opportunity to develop microbiome engineering approaches as a whole, to allow for application of biology-based control with microbial communities at the point of commissioning of engineered systems.

## General conclusions

- Interdisciplinary research collaboration is essential for *Legionella* spp. management, which overlaps expertise in microbiology, water quality, risk assessment, epidemiology, plumbing technology and engineering, building technology and engineering, and potentially other fields.
- Similarly, transdisciplinary collaborations and harmonization across the larger domains of science (research), government (legislation), and industry (application) are essential for the implementation of sensible, evidence-based *Legionella* spp. management and legislation.
- Regional differences in Legionnaires’ disease data driven by climate, legislation, network/building operation, reporting, human behavior, and so on, call for more international research collaborations and standardization to facilitate interregional reuse of the results.
- More in depth investigations of the ecology of *Legionella* spp. and their protist hosts in engineered aquatic ecosystems will help understanding microbial dynamics and aid decisions related to monitoring and alternative biological control strategies.
- Climate change is likely to impact multiple aspects of *Legionella* spp. risk including their concentrations in the environment, species diversity, and exposure pathways. It is imperative to identify and mitigate these risks proactively.
